# Phospholipase D1 is upregulated by vorinostat and confers resistance to vorinostat in glioblastoma

**DOI:** 10.1002/jcp.29882

**Published:** 2020-09-01

**Authors:** Dong Woo Kang, Won Chan Hwang, Yu Na Noh, Youra Kang, Younghoon Jang, Jung‐Ae Kim, Do Sik Min

**Affiliations:** ^1^ Department of Molecular Biology, College of Natural Science Pusan National University Busan Republic of Korea; ^2^ College of Pharmacy Yonsei University Incheon South Korea; ^3^ Institute for Innovative Cancer Research, Biomedical Research Center Asan Medical Center Seoul Republic of Korea; ^4^ College of Pharmacy Yeungnam University Gyeongsan South Korea; ^5^ Department of Biology and Chemistry Changwon National University Changwon Korea; ^6^ Present address: Dong Woo Kang, Medpacto Drug Development Research Center Medpacto Inc. Seoul Republic of Korea

**Keywords:** glioblastoma, HDAC inhibitor, phospholipase D1, resistance, tumorigenesis

## Abstract

Glioblastoma (GBM) is an aggressive brain tumor and drug resistance remains a major barrier for therapeutics. Epigenetic alterations are implicated in GBM pathogenesis, and epigenetic modulators including histone deacetylase (HDAC) inhibitors are exploited as promising anticancer therapies. Here, we demonstrate that phospholipase D1 (PLD1) is a transcriptional target of HDAC inhibitors and confers resistance to HDAC inhibitor in GBM. Treatment of vorinostat upregulates PLD1 through PKCζ‐Sp1 axis. Vorinostat induces dynamic changes in the chromatin structure and transcriptional machinery associated with *PLD1* promoter region. Cotreatment of vorinostat with PLD1 inhibitor further attenuates invasion, angiogenesis, colony‐forming capacity, and self‐renewal capacity, compared with those of either treatment. PLD1 inhibitor overcomes resistance to vorinostat in GBM cells intracranial GBM tumors. Our finding provides new insight into the role of PLD1 as a target of resistance to vorinostat, and PLD1 inhibitor might provide the basis for therapeutic combinations with improved efficacy of HDAC inhibitor.

## Graphical abstract

Phospholipase D1 (PLD1) is a transcriptional target of histone deacetylase (HDAC) inhibitors and confers resistance to HDAC inhibitor in glioblastoma (GBM). Cotreatment of vorinostat with PLD1 inhibitor further attenuates invasion, angiogenesis, colony‐forming capacity, and self‐renewal capacity, compared with those of either treatment. PLD1 inhibitor overcomes resistance to vorinostat in GBM cells and intracranial GBM tumors.

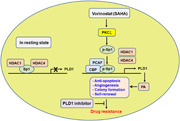

## 1 INTRODUCTION

Glioblastoma (GBM) is the most aggressive and lethal type of brain tumor that responds poorly to conventional treatment modalities (Wen & Kesari, 2008). Monotherapy has proven ineffective in treatment of GBM, likely due to the fact that GBM demonstrate multiple modes of resistance, including redundancy in prosurvival signaling pathways. Therefore, further work is urgently required to discover novel therapeutic targets and develop more effective combination strategies for GBM treatment. Epigenetic mechanisms are increasingly considered major factors contributing to the pathogenesis of cancer, including GBM (Kondo, Katsushima, Ohka, Natsume, & Shinjo, 2014). Aberrant loss of histone acetylation is a common feature in malignancy and epigenetic abnormalities in cancer cells could potentially be reversed by histone deacetylase (HDAC) inhibitor, which is known as effective therapeutic anticancer agents (Esteller, 2008; Falkenberg & Johnstone, 2014; Minucci & Pelicci, 2006). Various HDAC inhibitors such as vorinostat (suberoylanilide hydroxamic acid, SAHA) and valproic acid are currently being tested in clinical trials on GBM (Chinnaiyan et al., 2012; Friday et al., 2012; Galanis et al., 2009; Moroni et al., 2013). Although the use of HDAC inhibitor as monotherapy in the clinic has been validated in cutaneous T‐cell lymphoma, they are less effective against solid tumors (Lee, Kim, Kummar, Giaccone, & Trepel, 2008; Rasheed, Johnstone, & Prince, 2007). However, the mechanism of HDAC inhibitor resistance in solid tumors is not well‐elucidated and a better understanding will improve their clinical efficacy (Fantin & Richon, 2007). Accordingly, elucidation of resistance markers and its molecular mechanisms can lead to strategies to maximize the therapeutic efficacy of HDAC inhibitor by combining agents that target factor(s) associated with resistance. Phospholipase D (PLD) hydrolyzes phospholipid to generate phosphatidic acid, a lipid second messenger, and two isoforms of phosphatidylcholine‐specific PLD, PLD1 and PLD2 have been identified (Frohman, 2015). PLD is upregulated in various cancers and implicated in tumor malignancy, maintenance of self‐renewal of cancer stem cells, and resistance to radiotherapy and chemotherapy (Brown, Thomas, & Lindsley, 2017; Cheol Son et al., 2013; Kang, Choi, & Min, 2014; Kang et al., 2015; Kang, Lee, Hwang, 2017). PLD is known to increase the invasive migration and proliferation of GBM (Bruntz, Taylor, Lindsley, & Brown, 2014; O'Reilly et al., 2013; Sayyah et al., 2014). However, it is unknown whether PLD confers chemoresistance to GBM. In the present study, our goal was to investigate the effect of PLD1 on resistance to vorinostat in GBM and how vorinostat is responsible for the upregulation of PLD1.

## 2 MATERIALS AND METHODS

### 2.1 Cells and chemicals

U87MG (HTB‐14), U373 (HTB‐17), T98 (CRL‐1690), and U251‐MG (09063001) were obtained from the ATCC and the ECACC (Sigma‐Aldrich). Murine GL26 and GL261 cells were kindly provided by Prof H. Phillip Koeffler (University of California at Los Angeles) and Prof John R. Ohlfest (University of Minnesota), respectively. To establish the temozolomide (TMZ; 14163; Cayman Chemical)‐resistant cell lines, U251‐MG cells were exposed to a low dose of TMZ in culture media for 6 months and established TMZ‐resistant cells designated as U251‐TMZ‐R. IC_50_ for the growth inhibition of TMZ to U‐251MG and U251‐TMZ‐R are 21.6 and 271.3 mM, respectively. GBM‐PN‐528 and GBM‐MES‐83 were provided by Dr. D.H. Nam (Sungkyunkwan University). All chemicals used, if not indicated, were purchased from Cayman Chemical and Sigma‐Aldrich. Cells were irradiated at room temperature using γ‐rays a Cs‐137 blood irradiator (Eckert & Ziegler) at a dose rate of 6.0 Gy per min.

### 2.2 Transfection and luciferase reporter assays

Following the manufacturer's instructions, luciferase reporter of *PLD1* promoter (pGL4‐PLD1; Kang et al., 2008) and expression plasmids were transiently transfected into cells with Lipofectamine 3000 (Invitrogen) reagents. Relative luciferase activity was obtained by normalization of firefly and *Renilla* luciferase activity. Dual‐luciferase assay kits (E1910) were purchased from Promega.

### 2.3 Immunoprecipitation and western blot analysis

The following antibodies were used: anti‐α‐tubulin (sc‐8035), anti‐Sp1 (sc‐420), anti‐HDAC1 (sc‐81598), anti‐HDAC2 (sc‐9959), anti‐HDAC4 (sc‐46672), anti‐HDAC5 (sc‐133225), anti‐HDAC7 (sc‐74563), anti‐HDAC8 (sc‐17778), anti‐HDAC9 (sc‐398003), anti‐HDAC10 (sc‐393417), and anti‐PKCζ (sc‐17781; Santa Cruz Biotechnology) antibody; anti‐phospho ser/thr (#96315), anti‐active caspase‐3 (#9664), and acetyl‐histone 4 (#8647S; Cell Signaling) antibody. Rabbit polyclonal anti‐PLD antibody that recognizes both PLD1 and PLD2 was generated as described previously (Min et al., 2001). The signal densities on the blots were measured with ImageJ (Wayne Rasband) and normalized using anti‐α‐tubulin antibody.

### 2.4 Chromatin immunoprecipitation (ChIP) assay

ChIP assay was performed as previously described (Kang et al., 2015). The PLD1 promoter regions were amplified by polymerase chain reaction (PCR) using primers: 5′‐GGAGGCAGAA ATTCAGTTAT TGTAA‐3′ (forward), 5′‐AAGCAGCAGTCTATAAAATTGCATC‐3′ (reverse).

### 2.5 In vitro limiting dilution assay (LDA)

To determine the number of sphere‐forming units (SFU), in vitro LDA was performed as previously described (Kang et al., 2015). The average number of SFU counted upon replating of 10 LDAs derived from single spheres constituted the in vitro self‐renewal assay.

### 2.6 Colony‐forming assay

For colony formation assays, the cells were seeded into six‐well plates (2.5 × 10^4^ cells per well) and treated with the indicated agents in Dulbecco's modified Eagle's medium containing 10% fetal bovine serum. After 14 days, the cells were fixed in 4% paraformaldehyde in phosphate‐buffered saline for 10 min at room temperature and stained with 0.5% crystal violet in 20% methanol for 20 min. Images were captured using a flatbed scanner, and the cells were dissolved with 20% acetic acids in 20% methanol for 30 min.

### 2.7 PLD activity assay

PLD activity was assessed by measuring the formation of [^3^H]phosphatidylbutanol, the product of PLD‐mediated transphosphatidylation, in the presence of 1‐butanol as previously described (Kang, Lee, Hwang, et al., 2017).

### 2.8 Invasion assay

Invasion assays were performed as described previously (Kang et al., 2011). The extent of invasion, which was defined as movement of cells from the upper chamber to the lower chamber, was expressed as an average number of cells per microscopic field.

### 2.9 Apoptosis assay

Apoptotic cell death was measured by APC‐conjugated anti‐Annexin V Apoptosis Detection Kit I (550474; BD Bioscience). The terminal deoxynucleotidyl transferase dUTP nick‐end labeling (TUNEL) assay was performed using In Situ Cell Death Detection Kit, POD (Roche), according to the manufacturer's protocol.

### 2.10 Subcutaneous xenograft and intracranial tumor formation

Xenograft tumors were generated by subcutaneous injection of 1 × 10^7^ U87 cells. Tumors were measured with calipers to estimate their volumes. GBM cells were injected intracranially using a stereotactic device at a depth of 3 mm into the right forebrains of 9–10 weeks old athymic nude mice or syngeneic C57BL/6 mice (5 × 10^5^ cells/mouse). The mice were anesthetized with tribromoethanol (250 mg/kg; Sigma‐Aldrich) intraperitoneally. The mice were injected intraperitoneally with vorinostat (SML0061, 20 mg/kg; Sigma‐Aldrich), PLD1 inhibitor (VU0155069, 13206, 10 mg/kg; Cayman Chemical), or vorinostat/PLD1 inhibitor three times per week for 4 weeks. The protocol and procedures for animal studies were ethically reviewed and approved by the Institutional Animal Care Committee of Pusan National University.

### 2.11 Statistical analysis

Data were analyzed using Student's *t* test, and correlation coefficients were calculated using Spearman's *r*. Survival probability of mice bearing intracranial GBM cell lines, defined as the time from brain resection to death, was analyzed using Kaplan–Meier, and differences were evaluated using the log‐rank test. Statistical analysis was performed using GraphPad Prism 5.0 (GraphPad Software).

## 3 RESULTS

### 3.1 Vorinostat upregulates the expression of PLD1 via PKCζ signaling pathway

**Figure 1:**
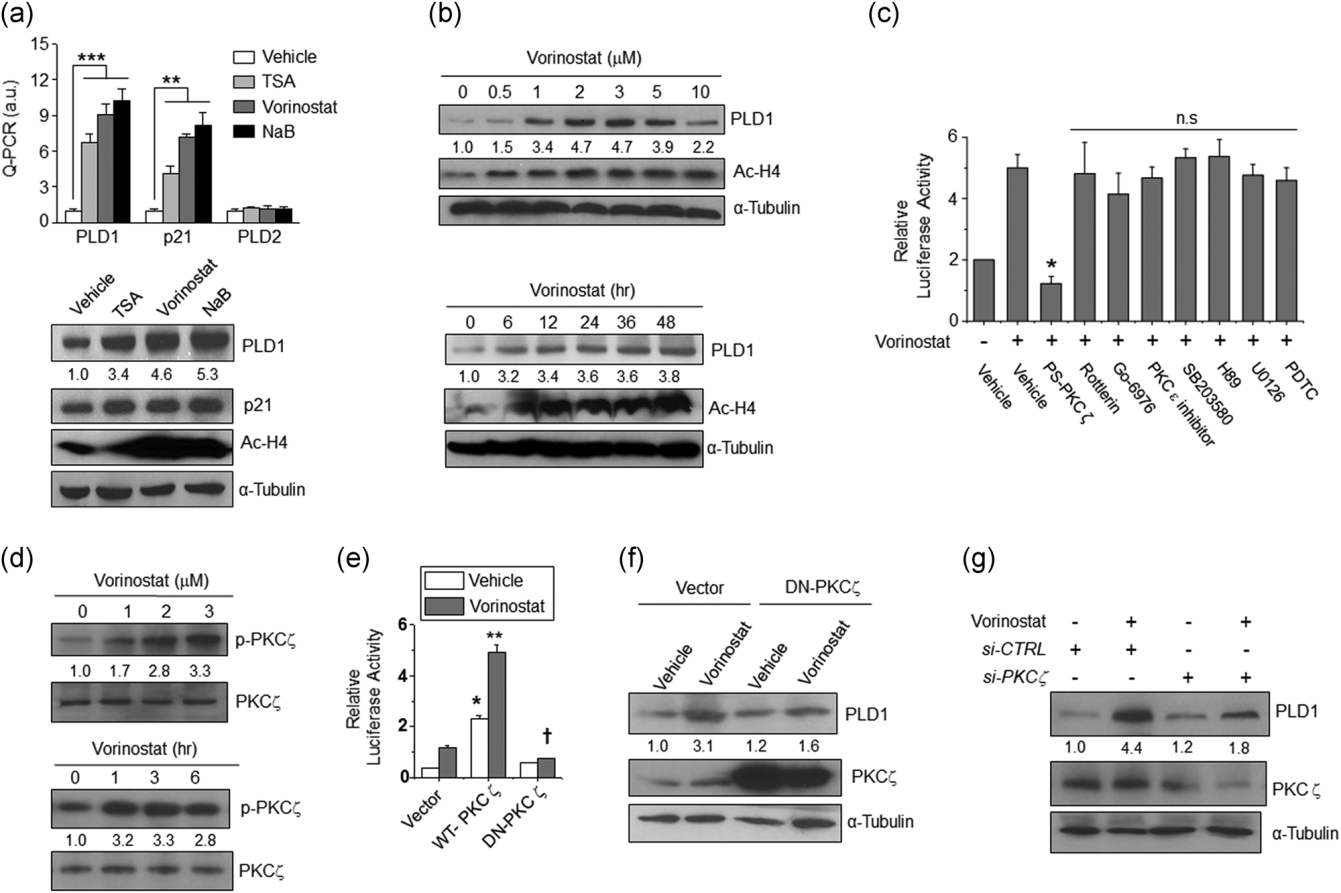
Vorinostat upregulates the expression of PLD1 via PKCζ signaling pathway. (a) U87 cells were treated with TSA (400 nM), vorinostat (2 μM), and NaB (5 mM) for 24 hr, and the expression of the indicated genes was analyzed by qPCR and western blot. (b) U87 cells were treated with the indicated concentration of vorinostat for 24 hr or 2 μM of vorinostat for the indicated time. The level of proteins was analyzed by western blot. (c) U87 cells were transfected with PLD1 promoter and pretreated with or without various inhibitors, PS‐PKCζ (50 μM), Rottlerin (5 μM), Go6976 (10 μM), PKCε V1‐2 (10 μM), SB203580 (20 μM), H89 (50 μM), U0126 (20 μM), or PDTC (50 μM) for 30 min, after which they were treated with vorinostat (2 μM) for 24 hr, followed by *PLD1* promoter activity assay. (d) Immunoblot analysis for vorinostat‐induced of phosphorylation of PKCζ. (e) Effect of wt or DN‐PKCζ on vorinostat‐induced *PLD1* promoter activity. (f) Effect of DN‐PKCζ on the vorinostat‐induced PLD1 expression. (g) Effect of PKCζ depletion on vorinostat‐induced PLD1 expression. The intensity of the indicated bands was normalized to the intensity of their respective α‐tubulin bands and quantified against each other. Results are representative of at least three independent experiments and shown as the mean ± *SEM*. *p < .05, **p < .01. NaB, sodium butyrate; ns, nonsignificant; PLD1, phospholipase D1; qPCR, quantitative polymerase chain reaction; TSA, trichostatin A; *SEM*, standard error of the mean

We investigated whether HDAC inhibitors affect the expression of PLD. Treatment with vorinostat, trichostatin A (TSA), or sodium butyrate (NaB) in U87MG cells, increased expression of PLD1 but not PLD2, as analyzed by quantitative PCR and western blot (Figure 1a). As a control, HDAC inhibitors increased the expression of p21 and acetylated histone 4 (Ac‐H4) (Figure 1a). HDAC inhibitor‐induced PLD1 upregulation was also observed in various human or murine GBM cells (Figure S1a,b). Vorinostat upregulated the expression of PLD1 in a dose‐ and time‐dependent manner in U87 cells (Figure 1b). These findings suggest that PLD1 but not PLD2 is a novel transcriptional target of HDAC inhibitors. To examine the putative participation of signaling molecules in vorinostat‐activated *PLD1* gene expression, *PLD1* promoter assay and immunoblot were carried out with U87 cells that had been pretreated with inhibitors of various signaling molecules before treatment with vorinostat (Figures 1c and S1c). This induction was largely abolished by PS‐PKCζ, an inhibitor of the atypical PKC, PKCζ, but not by other inhibitors (Figure 1c). Moreover, vorinostat induced the activation of PKCζ as indicated by the phosphorylation of PKCζ at threonine (Thr) 410 (Figure 1d), which is recognized to be critical for its activity (Standaert et al., 1999). Furthermore, the critical participation of PKCζ in vorinostat‐induced PLD1 upregulation was confirmed by reporter gene assay and immunoblot analysis (Figure 1e–g), which revealed that dominant negative (DN)‐PKCζ, a kinase‐inactive mutant form of PKCζ, and *PKCζ*‐directed small interfering RNA abrogated vorinostat‐induced PLD1 expression. Collectively, these results demonstrate that vorinostat upregulates the expression of PLD1 via PKC‐ζ at the transcriptional level.

### 3.2 Vorinostat induces marked Sp1 phosphorylation dependent on PKCζ, and Sp1 is required for vorinostat induction of PLD1

**Figure 2:**
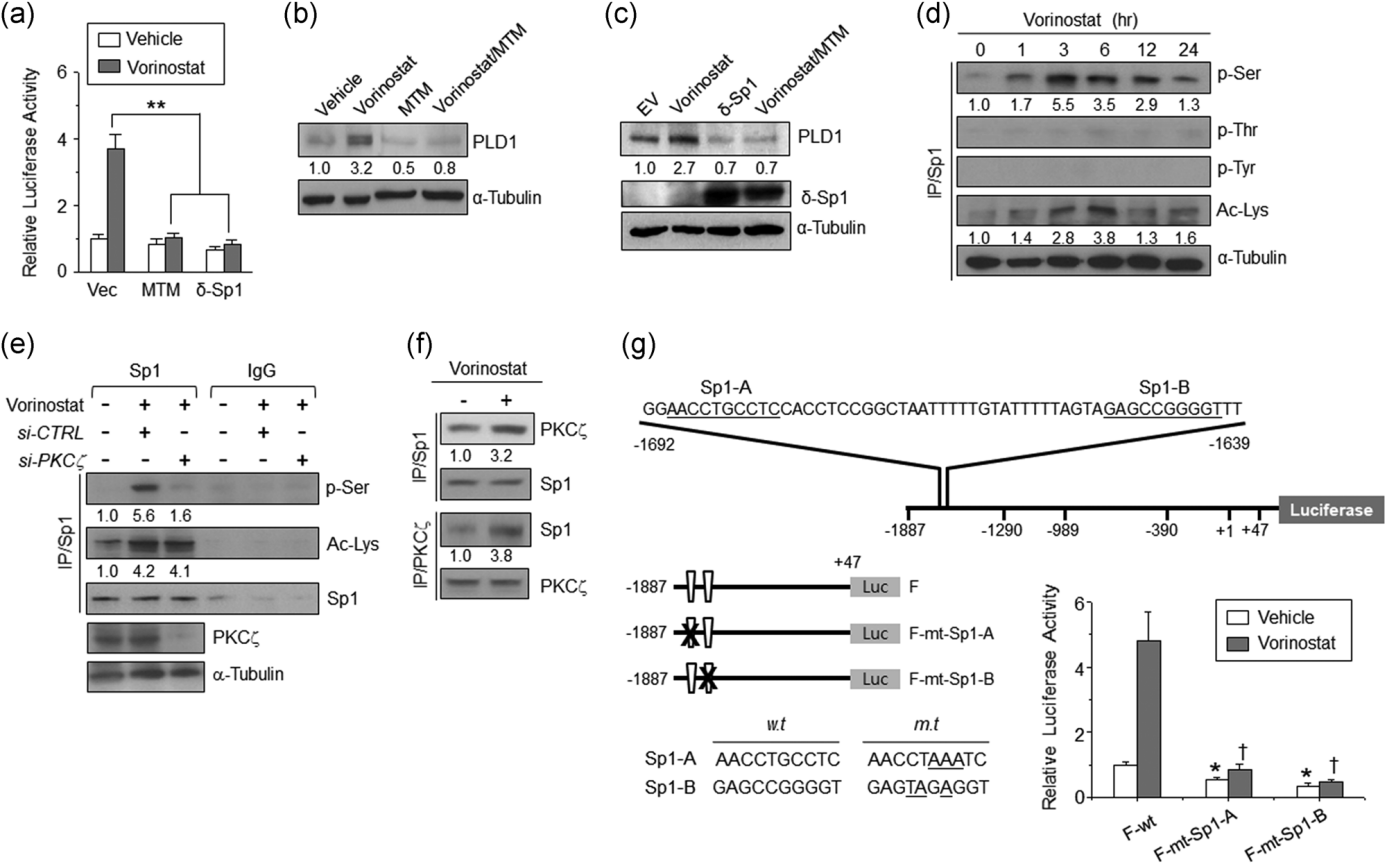
Vorinostat induces marked Sp1 phosphorylation dependent on PKCζ, and Sp1 is required for vorinostat induction of PLD1. (a) Effect of MTM or δ‐Sp1 on vorinostat‐induced *PLD1* promoter activity. (b) Effect of vorinostat and/or MTM on the expression of PLD1 protein in U87 cells. (c) Effect of vorinostat and/or δ‐Sp1on the expression of PLD1 protein. (d) Effect of vorinostat (2 μM) on serine‐, threonine‐, tyrosine‐phosphorylation, and acetylation of Sp1 in U87 cells. (e) Effect of PKCζ depletion on vorinostat‐induced phosphorylation and acetylation of Sp1. The cells were transfected with siRNA of PKCζ and treated with vorinostat. The lysates were immunoprecipitated and immunoblotted with the indicated antibodies. (f) Effect of vorinostat on the interaction between PKCζ and Sp1. (g) Schematic representation of putative Sp1‐binding sites and mutation of the Sp1‐binding sites present in PLD1 promoter (left panel). Effect of mutation of the Sp1‐binding sites on the promoter activity of PLD1 (right panel). The intensity of the indicated bands was normalized to the intensity of their respective α‐tubulin bands and quantified against each other. Results are representative of at least three independent experiments and shown as the mean ± *SEM*. *p < .05, **p < .01. MTM, mithramycin; PLD1, phospholipase D1; *SEM*, standard error of the mean; siRNA, small interfering RNA

Sp1‐dependent gene activation via HDAC inhibition has been observed (Gui, Ngo, Xu, Richon, & Marks, 2004; Yokota et al., 2004). Therefore, we examined whether vorinostat induction of PLD1 is mediated by Sp1 transcriptional factor using the pharmacologic inhibitor, mithramycin (MTM), which interferes with the binding of Sp1 to GC‐rich promoters, or δ‐Sp1, a dominant‐negative form of Sp1, which contains only DNA‐binding domain and interferes with Sp1 transactivation (Al‐Sarraj, Day, & Thiel, 2005; Blume et al., 1991). Pretreatment with MTM or overexpression of δ‐Sp1 dramatically inhibited vorinostat‐induced PLD1 expression (Figure 2a–c), suggesting that varinostat‐mediated PLD1 upregulation is Sp1‐dependent. We further examined whether PKCζ exerted vorinostat‐activated *PLD1* gene expression through Sp1. Vorinostat caused marked Sp1 phosphorylation at serine residues, whereas no phosphorylation of Sp1 was observed at the threonine or tyrosine residues (Figure 2d). Vorinostat also induced acetylation of Sp1 (Figure 2d). Moreover, vorinostat‐induced serine phosphorylation of Sp1, but not acetylation, was PKCζ dependent because depletion of PKCζ suppressed vorinostat‐induced serine phosphorylation of Sp1 (Figure 2e). The activated form of PKCζ (phosphorylated at Thr 410) not only exists in the cytosol, but is also present in the nucleus, strengthening the concept that Sp1 could be a nuclear target of PKC (Zhang, Liao, & Dufau, 2006). Moreover, Sp1 interacts with PKCζ, and vorinostat increased the association between Sp1 and PKCζ (Figure 2f). We found two putative Sp1‐binding sites in the *PLD1* promoter (Figure 2g). The reporter gene assay using a series of 5΄‐deletion constructs of the *PLD1* promoter showed that the region from −1,887 to −1,290, which contains two putative Sp1‐binding sites, is involved in vorinostat‐mediated *PLD1* promoter activation (Figure S2). As a positive control, the expression of Sp1 reporter gene was enhanced by vorinostat. Moreover, mutation of two Sp1‐binding sites, Sp1‐A or Sp1‐B significantly attenuated vorinostat‐induced *PLD1* promoter activity, respectively (Figure 2g). Taken together, these results suggest the functional importance of the PKCζ‐Sp1 signaling axis in the vorinostat‐induced induction of PLD1.

### 3.3 Vorinostat elicits the dissociation of HDAC1 and HDAC4 and the recruitment of CREB‐binding protein/p300/CBP‐associated factor (CBP/PCAF) and Sp1 onto the PLD1 promoter

**Figure 3:**
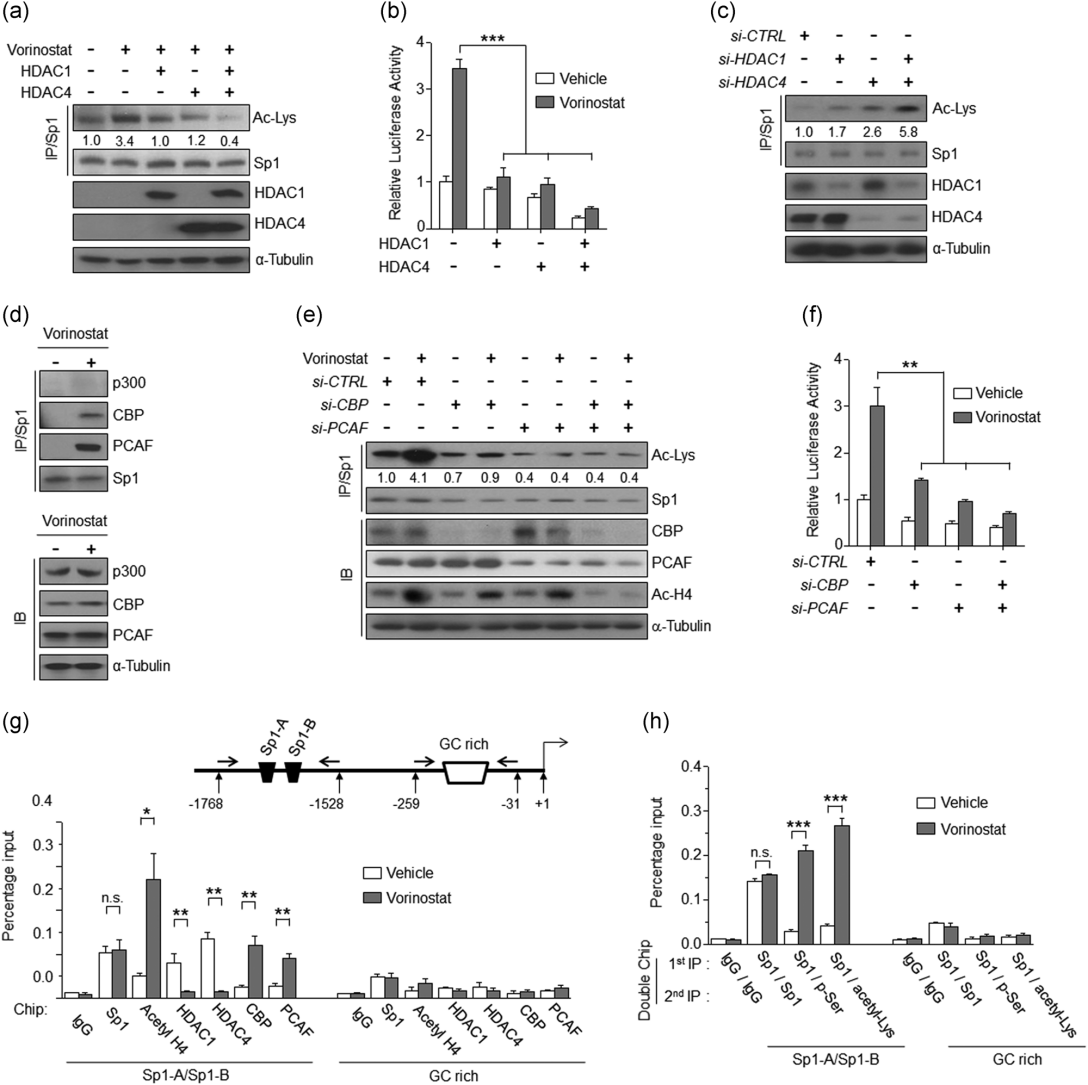
Vorinostat elicits the dissociation of HDAC1 and HDAC4 and the recruitment of CBP/PCAF and Sp1 onto the PLD1 promoter. (a) Effect of overexpression of HDAC1 and HDAC on the acetylation of Sp1 in U87 cells. (b) Effect of HDAC1 and HDAC4 on the activity of *PLD1* promoter. (c) Effect of depletion of HDAC1 and HDAC4 on the acetylation of Sp1 (d) Effect of vorinostat on the interaction of Sp1 with various HAT. (e) Effect of depletion of CBP and PCAF on the acetylation of Sp1 in U87 cells. (f) Effect of depletion of CBP and PCAF on the activity of *PLD* promoter (g‐h) ChIP assays for binding of the indicated proteins to *PLD1* promoters in U87 cells treated with vorinostat (2 μM) for 6 hr, after which a single or double ChIP assay was performed using the indicated antibodies. The GC‐rich region of the PLD1 promoter was used as a control. The intensity of the indicated bands was normalized to the intensity of their respective α‐tubulin bands and quantified against each other. Results are representative of at least three independent experiments, and shown as the mean ± *SEM*. *p < .05; **p < .01; ***p < .001. CBP; CREB‐binding protein; ChIP, chromatin immunoprecipitation; HAT, histone acetyltransferase; HDAC, histone deacetylase; PCAF, p300/CBP‐associated factor; PLD1, phospholipase D1; n.s., not significant; *SEM*, standard error of the mean

We next investigated whether vorinostat affects the association of Sp1 with HDAC or histone acetyltransferase (HAT). In U87 cells, Sp1 was constitutively associated with HDAC1, 2, or 4, but not HDAC5, 7, 8, 9, or 10, and vorinostat reduced the interaction of Sp1 with HDAC1 and HDAC4, but not HDAC2 (Figure S3). Moreover, ectopic expression of HDAC1 and 4 decreased both vorinostat‐induced Sp1 acetylation and *PLD1* promoter activity (Figure 3a,b), whereas depletion of HDAC1 and HDAC4 increased the acetylation of Sp1 (Figure 3c). Furthermore, vorinostat increased the association of Sp1 with HAT, CBP, or PCAF as a transcriptional coactivator, but not with p300 (Figure 3d). Depletion of CBP and PCAF reduced both vorinostat‐induced Sp1 acetylation and *PLD1* promoter activity (Figure 3e,f). Unexpectedly, vorinostat did not affect binding of Sp1 to the *PLD1* promoter (Figure 3g). Vorinostat induced hyperacetylation of the histone H4 associated with the *PLD1* promoter region through association with PCAF and CBP, as well as the dissociation of HDAC1 and 4 (Figure 3g). As a negative control, vorinostat did not affect the GC‐rich region (−259 to −31 nucleotides) on the *PLD1* promoter. Interestingly, double ChIP assay revealed that vorinostat significantly enhanced the binding of both phosphorylated and acetylated Sp1 to the *PLD1* promoter region (Figure 3h). Taken together, our results show that vorinostat induces dynamic changes in the chromatin structure and transcriptional machinery associated with the *PLD1* promoter region, which results in transcription of the *PLD1* gene through Sp1 acetylation, dissociation of HDAC1/4, and recruitment of CBP/PCAF onto the *PLD1* promoter.

### 3.4 Combination of vorinostat with depletion or inhibition of PLD1 promotes cell death of GBM

**Figure 4:**
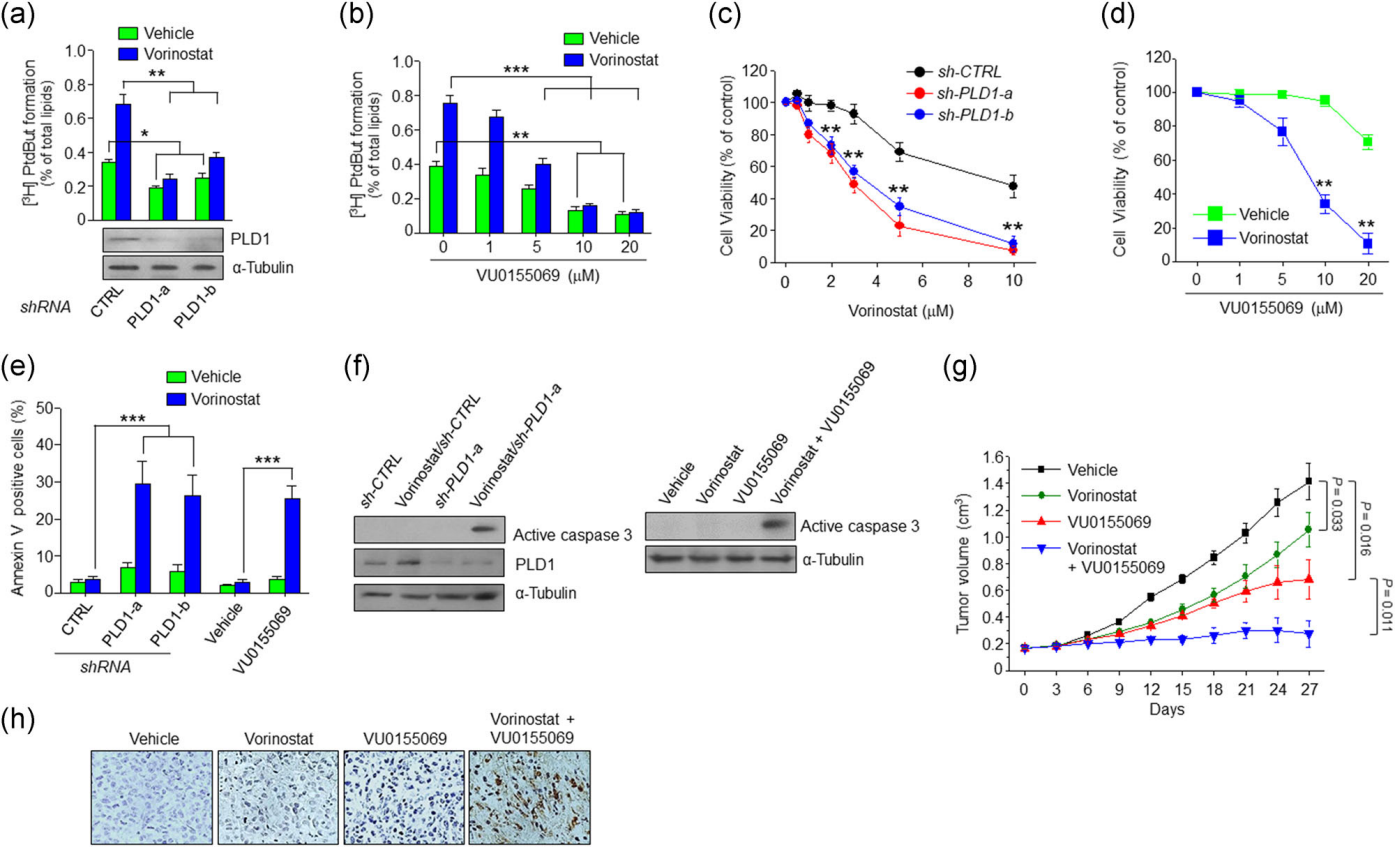
Combination of vorinostat with depletion or inhibition of PLD1 promoted cell death of GBM. (a) U87 cells were transfected with two kinds of shRNA of PLD1 (−a, −b), treated with vorinostat (2 μM) for 6 hr, and then PLD activity was measured. (b) The cells were treated with the indicated drugs and then PLD activity assay was performed. Effect of PLD1 depletion (c) and PLD1 inhibitor (d) in the presence or absence of vorinostat on the viability of U87 cells as analyzed by MTT assay. (e) Effect of depletion and inhibition of PLD1 in the absence or presence of vorinostat on the apoptosis as analyzed by Annexin V assay. Annexin V‐positive cells were quantified. (f) Effect of depletion (left) and inhibition (right) of PLD1 in the absence or presence of vorinostat on the level of the active caspase 3 as analyzed by western blot. (g) Athymic nude mice were injected subcutaneously with U87 cells (*n* = 7/group). Mice were subjected to intraperitoneal injection with vehicle, VU0155069 (10 mg/kg) alone, vorinostat (20 mg/kg) alone, or in combination three times a week for 27 days. The tumor volume of mice was measured with vernier calipers every 3 days. (h) The paraffin‐embedded tumor sections were analyzed by TUNEL assay. Results are representative of at least three independent experiments, and shown as the mean ± *SEM*. *p < .05; **p < .01; ***p < .001. GBM, glioblastoma; MTT, 3‐(4,5‐dimethylthiazol‐2‐yl)‐2,5‐diphenyltetrazolium bromide; n.s., not significant; PLD1, phospholipase D1; *SEM*, standard error of the mean; shRNA, short hairpin RNA; TUNEL, terminal deoxynucleotidyl transferase dUTP nick‐end labeling

We next examined whether vorinostat‐induced PLD1 expression is responsible for increased PLD activity. As shown in Figure 4a, vorinostat stimulated the enzymatic activity of PLD, which was inhibited by PLD1 depletion using two kinds of short hairpin RNA. Moreover, vorinostat‐induced PLD activation was decreased by PLD1 inhibitor, VU0155069 (Figure 4b). Thus, it is suggested that vorinostat‐induced PLD1 expression is associated with increased PLD activity. As PLD is known to protect anticancer drug‐induced cell death (Kang et al., 2014), we further examined whether vorinostat‐induced PLD1 expression is associated with chemoresistance. Vorinostat below 2 μM did not affect the viability of U87 cells but vorinostat above 3 μM reduced the viability of the glioma cells (Figure 4c). A combination of varinostat with depletion or inhibition of PLD1 significantly decreased the viability of U87 cells, compared with that of either treatment (Figure 4c,d). Moreover, a combinational treatment of vorinostat (2 μM) with PLD1 depletion or PLD1 inhibitor significantly promoted apoptosis of U87 cells, relative to that of either treatment as analyzed by Annexin V assay (Figure 4e). Moreover, the combination further increased the levels of active caspase‐3 and the population of subG1 apoptotic cells compared with that of either treatments (Figures 4f and S4a), suggesting that the combinational therapy promotes cell death. To further evaluate the anticancer activity of the combinational therapy, we examined the effect of either alone or combination on the growth of U87 GBM cells subcutaneously implanted in nude mice. Vorinostat or PLD1 inhibitor alone inhibited the growth of tumors (Figure 4g). The combination of the two drugs was more effective at reducing tumor formation than being used alone. Furthermore, the combined treatment in the mice promoted apoptosis as analyzed by TUNEL assay (Figure 4h). Collectively, these results suggest that combined treatment of vorinostat with PLD1 inhibition promotes cell death of GBM.

### 3.5 Combinational treatment of vorinostat with PLD1 inhibitor further suppresses invasion and angiogenesis

**Figure 5:**
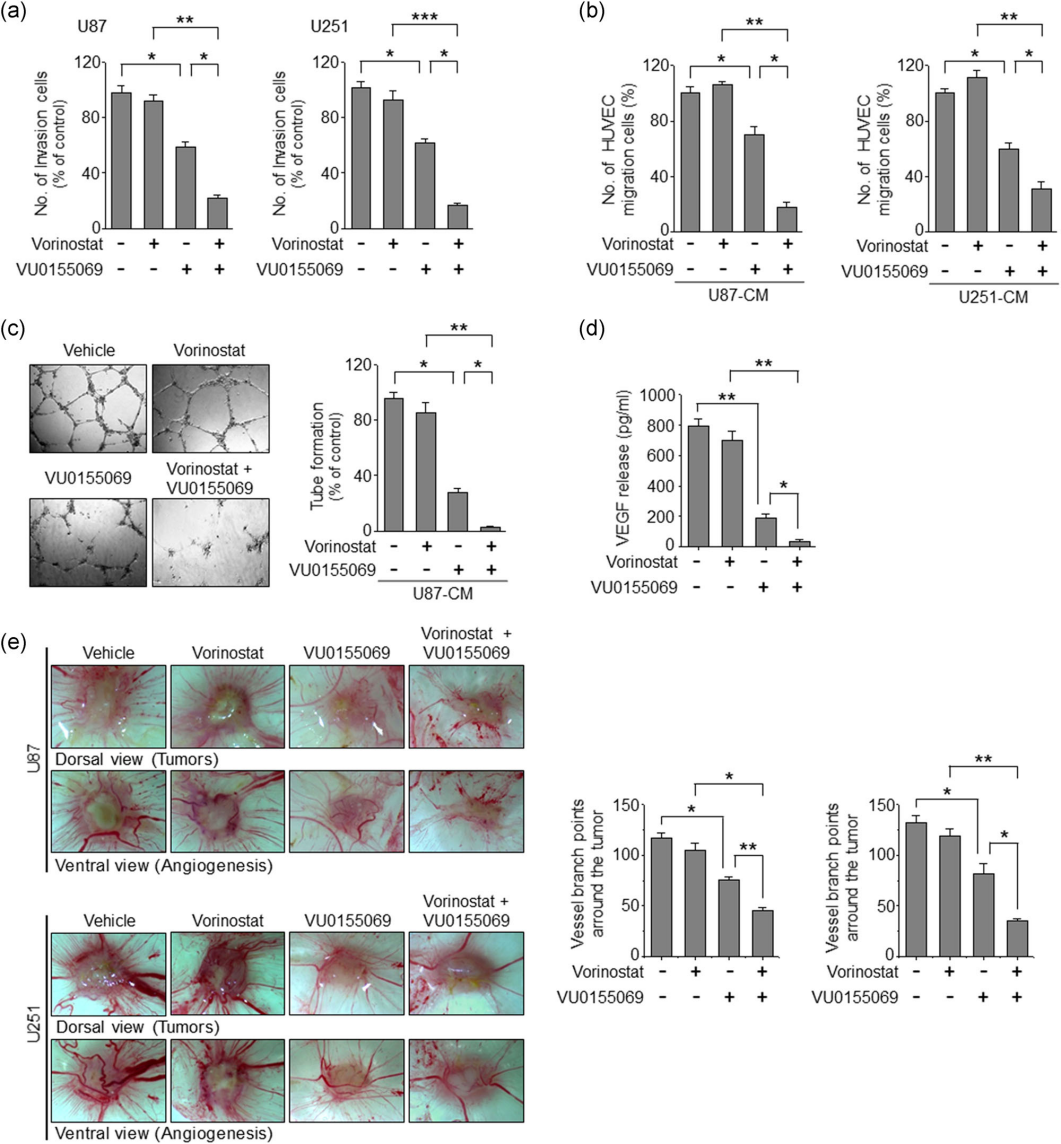
Combinational treatment of vorinostat with PLD1 inhibitor suppresses invasion and angiogenesis. (a) The cells were seeded in matrigel‐coated invasion chambers and treated with vorinostat (2 μM) and/or VU0155069 (10 μM) for 24 hr. The extent of invasion was expressed as an average number of cells per microscopic field. The cells were treated with the indicated drug (s) for 36 hr. Conditioned medium was collected and applied to HUVEC, and then migration (b) and tube formation (c) were measured. (d) U87 cells were treated with the indicated drug(s) for 36 hr and then secretion of VEGF was quantified by ELISA. (e) Inhibitory effects of PLD1 inhibitor and vorinostat on angiogenesis in U87 and U251 CAM‐implanted tumors. After the cells were loaded (1.5 × 10^6^ cells/CAM) onto CAMs, the indicated drug(s) were administered at the time of implantation. Five days after implantation, CAM were resected and imaged under the microscope. Tumor vasculature and the number of vessels were analyzed. The data represent the mean ± *SEM* of at least six chick embryos. Results are representative of at least three independent experiments, and shown as the mean ± *SEM*. *p < .05; **p < .01; ***p < .001. ELISA, enzyme‐linked immunosorbent assay; HUVEC, human umbilical vein endothelial cells; PLD1, phospholipase D1; *SEM*, standard error of the mean; VEGF, vascular endothelial growth factor

We further investigated the combinational therapeutic effect of vorinostat and PLD1 inhibitor against invasion and angiogenesis. Treatment of vorinostat did not affect the invasion of U87 and U251 cells, but PLD1 inhibitor significantly suppressed the invasion of GBM cells (Figure 5a). Cotreatment further decreased the invasive capacity of GBM cells compared with either of the treatment alone (Figure 5a). The drug(s) were treated in the GBM cells, after which conditioned media were applied to human umbilical vein endothelial cells (HUVEC) for migration and angiogenic assay. PLD1 inhibitor but not vorinostat, significantly decreased the migration and tube formation of HUVEC, an important feature of angiogenesis (Figure 5b,c). The combined treatment further suppressed the migration and tube formation of HUVEC compared with those of either one. Additionally, PLD1 inhibitor but not vorinostat, significantly decreased the production of vascular endothelial growth factor (VEGF) in U87 cells (Figure 5d). Combined treatment further inhibited the release of VEGF compared with either treatment. To further verify the antiangiogenic or antitumorigenic effects of these drugs, we implanted U87 and U251 cells into Chick chorioallantoic membrane (CAM), respectively. The implantation of cancer cells in CAM increased the number of newly formed blood vessels, which was assessed based on vessel branch points. Such tumor‐induced neovascularization was significantly suppressed by treatment with PLD1 inhibitor. Vorinostat had a marginal effect on the tumor‐induced neovascularization. The combined treatment significantly suppressed neovascularization when compared with monotherapy (Figure 5e). Therefore, the potential anticancer efficacy of the combined treatment with vorinostat and PLD1 inhibitor regimens is linked to inhibitory effects against invasion, migration, and angiogenesis.

### 3.6 Combinational therapy of vorinostat with PLD1 inhibitor efficiently attenuates the tumorigenic potential of GBM

**Figure 6:**
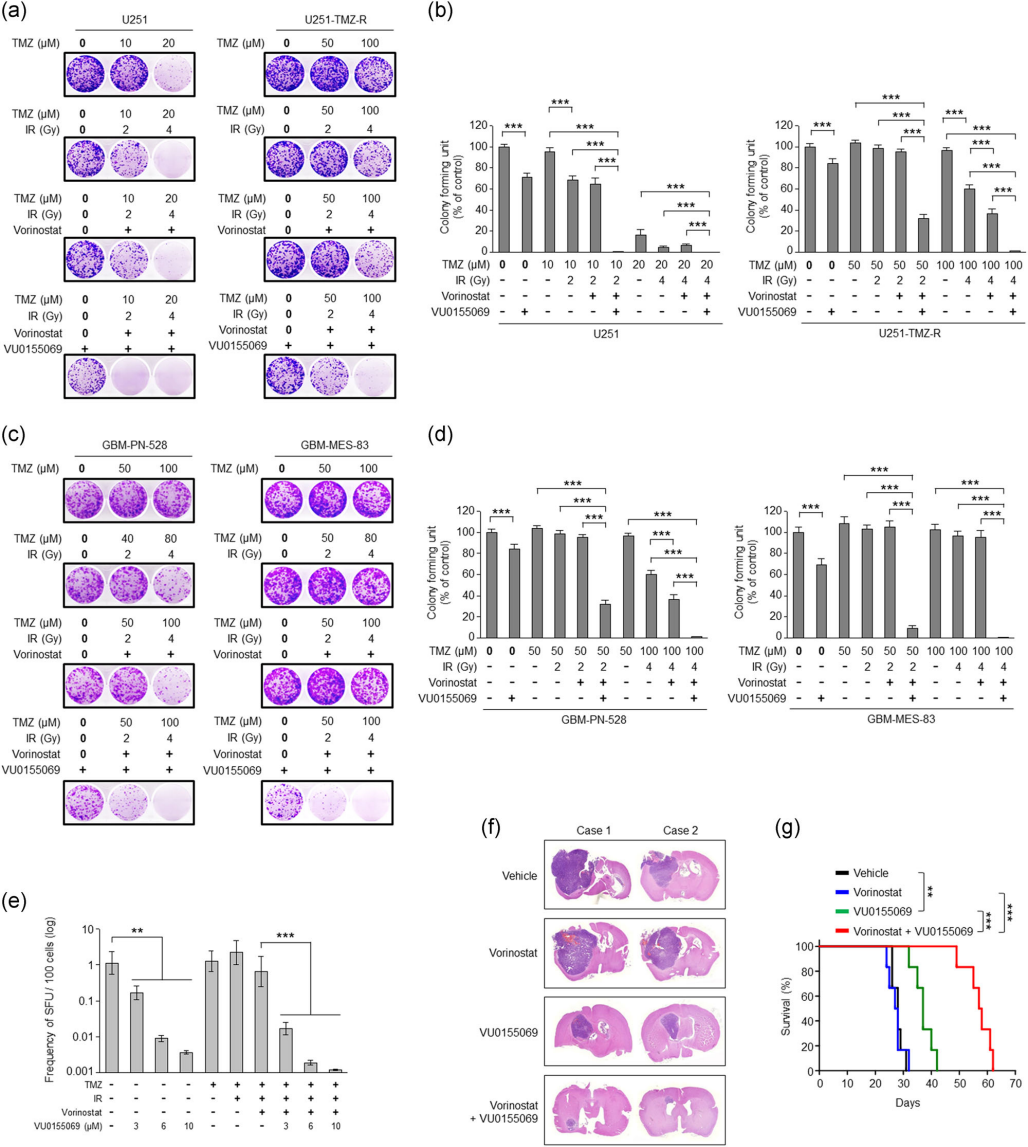
Combinational therapy of vorinostat with PLD1 inhibitor efficiently attenuates tumorigenic potential of GBM. (a) Effect of PLD1 inhibitor, vorinostat, TMZ, and IR on the colony‐forming capacity in U251 and U251‐TMZ‐R. (b) Quantification of colony formation. (c) Effect of PLD1 inhibitor, vorinostat, TMZ, and IR on the colony formation in GBM‐PN‐528 and GBM‐MES‐83. (d) Quantification of colony‐forming capacity in (c). (e) Frequency of sphere‐forming units (SFU) of GBM‐MES‐83 cells as determined by in vitro limiting dilution assay under the treatment with the indicated drugs and/or IR. (f) GL26 cells (*n* = 6/group) were intracranially transplanted into the brains of immunocompromised mice and then treated intraperitoneally with vehicle, VU0155069 (10 mg/kg) alone, vorinostat (20 mg/kg) alone, or in combination three times a week for 4 weeks. Representative images of H&E‐stained sections of mouse brains. (g) Survival of mice was evaluated (*n* = 6/group, Kaplan–Meier model with two‐sided log‐rank test). **p < .01; ***p < .001. Results are representative of at least three independent experiments, and shown as the mean ± *SEM*. **p < .01; ***p < .001. GBM, glioblastoma; H&E, hematoxylin and eosin; IR, ionizing radiation; MES, mesenchymal; PLD1, phospholipase D1; *SEM*, standard error of the mean; TMZ, temozolomide

Combination with alkylating drug, TMZ, and ionizing radiation (IR) is currently used as a standard treatment for GBM. We tried to investigate whether PLD1 inhibitor or vorinostat affect the standard chemoradiotherapies in GBM and TMZ‐resistant GBM. As analyzed by colony‐forming capacities, U251‐TMZ‐R cells showed resistance to TMZ, TMZ/IR, or TMZ/IR/vorinostat, relative to U251 cells (Figure 6a,b). PLD1 inhibitor only treatment significantly suppressed colony‐forming capacities in both U251 and U251‐TMZ‐R, and combination of TMZ/IR/vorinostat with PLD1 inhibitor markedly reduced the colony‐formation, compared with that of TMZ/IR/vorinostat in U251‐TMZ‐R (Figure 6a,b). We further investigated the therapeutic effect in patient‐derived GBM cell lines. Genome‐wide transcriptome analyses have suggested that GBM can be divided into four clinically relevant subtypes: classic, mesenchymal (MES), neural and proneural (PN) GBM (Phillips et al., 2006; Verhaak et al., 2010). We examined the colony‐forming capacities using MES and PN subtype of GBM. GBM‐PN‐528 (PN subtype GBM) and GBM‐MES‐83 (MES subtype GBM) showed more resistance to TMZ at higher concentration (50 and 100 μM) (Figure 6c,d). Combination of TMZ/IR or TMZ/IR/vorinostat showed resistance in GBM‐MES‐83, relative to GBM‐PN‐528. Actually, MES subtype of GBM is known to be more aggressive and radio‐resistant than PN subtype of GBM (Mao et al., 2013). PLD1 inhibitor alone significantly suppressed the colony formation, and a combination of TMZ/IR/vorinosta with PLD1 inhibitor dramatically abolished the colony‐forming capacities compared with that of TMZ/IR/vorinostat (Figure 6c,d). Moreover, PLD1 inhibitor reduced the mean sphere‐forming capacity of the GBM‐MES‐83 cells by an average of 400‐fold, based on *in vitro* limiting dilution assay (Figure 6e). Although TMZ, TMZ/IR, or TMZ/IR/vorinostat did not affect the sphere‐forming capacity of GBM‐MES‐83 cells, a combination of TMZ/IR/vorinosta/PLD1 inhibitor significantly suppressed the mean sphere‐forming capacity of GBM compared with that of PLD1 inhibitor alone (Figure 6e). Thus, it is suggested that PLD1 contributes to the self‐renewal capacity of GBM. In addition, PLD1 inhibitor significantly suppressed the expression of stemness‐related genes (CD44, CD133, Bmi‐1, and ALDH1A1) under sphere‐culture condition of MES‐83 cells (Figure S5a). In addition, PLD1 expression was significantly correlated with the levels of stemness‐related factors as assessed in the TCGA GBM expression profile database (Brennan et al., 2013; Figure S5b). Furthermore, we investigated the effect of the drug(s) on the tumor‐propagating capacity of GBM using orthotopic model. Mouse GL‐26 GBM were transplanted into the brains of syngeneic C57BL/6 mice, and the treatment of PLD1 inhibitor reduced tumor formation and significantly increased survival (Figure 6f,g). Treatment with TMZ had a marginal effect on both tumor formation and survival. Combinational treatment remarkably suppressed the intracranial tumor formation and increased survival compared with that of PLD1 inhibitor alone. Collectively, these results suggest that targeting PLD1 overcome chemoradiotherapeutic resistance and PLD1 inhibitor might provide the basis for therapeutic combinations with improved clinical efficacy of HDAC inhibitor in GBM.

## 4 DISCUSSION

**Figure 7:**
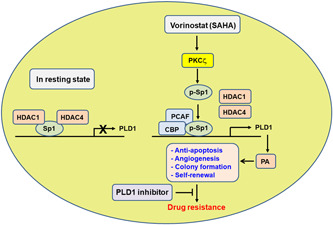
Schematic diagram representing the mechanism of the resistance to vorinostat in GBM. HDAC1 and HDAC4 bind to Sp1 and thus repress expression of PLD1. Vorinostat activates PKCζ and induces phosphorylation of Sp1, dissociation of HDAC1/4, and recruitment of CBP/PCAF onto the *PLD1* promoter, followed by upregulation of PLD1. PA generated by PLD1 is responsible for antiapoptosis, angiogenesis, colony formation, and self‐renewal capacity, followed by resistance to vorinostat in GBM. CBP, CREB‐binding protein; GBM, glioblastoma; HDAC, histone deacetylase; PA, phosphatidic acid; PCAF, p300/CBP‐associated factor; PLD1, phospholipase D1

In the present study, we demonstrate that PLD1 acts as a novel transcriptional target of HDAC inhibitors and confers resistance to vorinostat in GBM. GBM, a very aggressive brain tumor remains one of the deadliest of malignancies, with limited treatment options and a high rate of recurrence, and thus represents an urgent unmet medical need (Wen & Kesari, 2008). GBM recurrence is linked to the epigenetic mechanisms and cellular pathways (Esteller, 2008). Consequently, multidisciplinary research efforts, including epigenetic modalities—HDAC inhibitors such as vorinostat, are certainly needed. HDAC inhibitors are epigenetic agents that target the aberrant epigenetic characteristics of the tumor cells. However, molecular determinants of resistance to HDAC inhibitors are poorly understood. A better understanding of the mechanisms that determine resistance to HDAC inhibitors would provide the basis for therapeutic combinations with improved clinical efficacy. PLD has been reported to be intimately associated with the signaling pathways modified in GBM (Bruntz et al., 2014; Colman et al., 2003; Kang et al., 2014; Mathews et al., 2015). Moreover, PLD1 inhibitor exhibits potent anticancer activity in a patient‐derived xenograft model harboring *APC* tumor suppressor and *PI3KCA* mutation, which results in hyperactivation of the mitogenic Wnt/β‐catenin and PI3K/Akt signaling pathways (Kang, Lee, Suh, et al., 2017), suggesting that inhibition of PLD1 might overcome limited clinical benefit due to drug resistance. Therefore, our finding of HDAC‐induced PLD1 upregulation led to investigate the possibility of PLD1 as a new resistance target of HDAC inhibitor and effective combination strategies for GBM treatment. The changes in chromatin structure might provide a permissive state of the *PLD1* promoter. Multiple layer of regulation, including PKCζ‐induced Sp1 phosphorylation, histone acetylation of the *PLD1* gene promoter, and release of the inhibitory HDAC, and recruitment of a transcriptional coactivator complex, account for vorinostat‐induced *PLD1* upregulation (Figure 7). PLD1 inhibitor or vorinostat alone has a marginal effect on apoptosis, but the combination of these agents potentiated the proapoptotic efficacy in GBM. Recently we have reported PLD as a new player in the molecular machinery regulating autophagy (Jang, Choi, & Min, 2014). The role of autophagy in cancer and treatment responsiveness is undoubtedly complicated. PLD1 inhibition augments the efficacy of anticancer regimens via facilitation of autophagic pathways (Jang et al., 2014). The control of autophagy might also be used as a therapeutic strategy to treat cancer cells that are resistant to cell death. Our findings show that vorinostat‐induced PLD1 upregulation plays a pivotal role in protection from apoptosis. Furthermore, combination of the drugs significantly suppressed invasion, angiogenesis, colony formation, self‐renewal capacity of GBM, and intracranial GBM tumor formation. PLD1 inhibition overcame resistance to conventional therapeutic treatment of GBM. As cancer stem cells contributes to drug resistance, targeting PLD1 effectively might overcome GBM‐mediated therapeutic resistance. As PLD1 is a new target of vorinostat resistance, and combinational therapy of PLD1 inhibitor with vorinostat might be a potential therapeutic strategy against GBM tumorigenesis, it would be interesting to know whether it is possible to develop some biomarkers of therapeutic efficacy that could facilitate a more precise selection of the most suitable candidates for innovative combination therapy.

## CONFLICT OF INTERESTS

The authors declare that there are no conflict of interests.

## AUTHOR CONTRIBUTIONS

D. W. K., W. C. H., J. K., and D. S. M. contributed to the design of study protocol. D. W. K., W. C. H., Y. N. N., and Y. K. performed the experiment. D. S. M. conceived and designed the study. D. W. K., J. K., Y. J., and D. S. M. contributed to data analysis. D. W. K., J. K., Y. J., and D. S. M. wrote the manuscript. D. S. M. supervised the research. All authors read and approved the final version of the manuscript.

## Supporting information

Supporting information

## Data Availability

The data that support the findings of this study are available from the corresponding author D.S.M. upon reasonable request.
