# Supplementary material for: Phospholipase D1 is upregulated by vorinostat and confers resistance to vorinostat in glioblastoma
Source: J Cell Physiol. 2020 Sep 1;236(1):549–60. doi: 10.1002/jcp.29882 (PMC7692931; doi:10.1002/jcp.29882)
Supplement: Supplementary file 1 — Supporting information [file JCP-236-549-s001.docx]

**SUPPLEMENTARY INFORMATION**

**1. Supplementary materials and methods**

***1.1 q-PCR***

The following primers were used: For PLD1, Sense 5´- TGT CGT GAT ACC ACT TCT GCC A -3´; antisense 5´- AGC ATT TCG AGC TGC TGT TGA A -3´ For PLD2, sense 5´- CAT CCA GGC CAT TCT GCA C -3´; antisense 5´- GTG CTT CCG CAG ACT CAA GG -3´ For β-actin, sense 5´- GTG GTC TCC TCT GAC TTC AAC -3´; antisense 5´- TCT CTT CCT CTT GTG CTC TTG -3´

***1.2 Small interfering RNA***

siRNA of two independent 21-nucleotide sequences correspondingto human PLD1 sequences (PLD1-a nucleotides: 1571 to 1591, AAGGUGGGACGACAAUGAGCA; PLD1-b nucleotides: 3031 to 3051, AGGACATTCAGGATCCAGTGA) and control siRNA were purchasedfrom Dharmacon Research Inc (Lafayette, Colo).

***1.3 Site directed mutagenesis***

The mutations in the putative Sp1 binding sites were generated by site-directed mutagenesis using the Quick Change Site-Directed Mutagenesis Kit (Stratagene, LaJolla, CA), according to the manufacturer's instructions. For the point mutation of Sp1 sites located from -1690 to -1681 and from -1650 to -1641, the following primers were used: mt1Sp1 sense 5’ AGCTTCGATT ACAGGAACCTAAATCCACCTCCGGCTA 3’; antisense 5’ TAGCCGGAGG TGGATTTAGGTTCCTGTAATCGAAGCT 3’; mt2 Sp1 sense 5’ GTATTTTTAG TAGAGTAGAGGTTTCGCCATGTGGCCC AG 3’; antisense 5’ CTGGGCC ACATGGCGAA ACCTCTACTCTACTA AAAATAC 3’

***1.4 Cell viability assay***

For the cell viability assay, a 3-(4, 5-dimethylthiazol-2-yl)-2,5-diphenyltetrazolium bromide assay was performed. Absorbance was measured using the ELISA reader at 540 nm, and the percentage of viability was indicated relative to the control.

***1.5 Flow cytometry***

Cells were collected at the density of 2x10^6^ and fixed 80% absolute ethanol for overnight. After fixation, cells were stained with propidium idodide (1ug/ml) and then analyzed using a FACScan flow cytometry. Cell cycle analysis was performed by flow cytometer using a commercially available software package (Modifit; Verity Winlist, Topsham, ME).

***1.6 In vitro tube formation***

Human umbilical vein endothelial cells (HUVEC) were then seeded on the Matrigel-coated wells, cultured at 37°C in a 5% CO_2_ atmosphere incubator for 12 ~ 24 h, and observed with a light microscope equipped with a digital CCD camera to verify the formation of the capillary-like structures.

***1.7 Chick chorioallantoic membrane (CAM) model of angiogenesis***

The CAM model of angiogenesis was assessed according to the previously published methods ([Colman et al., 2003](#_ENREF_1)). Digital images of the CAM sections underneath the filters were collected, using a digital image analyzer (DMI-300, DMI). The images were then analyzed using Image-Inside software. The number of vessel branch points contained in a circular region (equal to the area of each filter disk) was counted. One image was counted for each CAM preparation, and findings from 6 to 8 CAM preparations were analyzed for each of the treatment conditions. The resulting angiogenesis index was the mean ± SEM of the new branch points for each set of samples. The number of vessel branch points contained in a tumor region was counted by two observers in a double-blind manner.

**REFERENCES**

Colman RW, Pixley RA, Sainz IM, Song JS, Isordia-Salas I, Muhamed SN, Powell JA, Jr., Mousa SA. 2003. Inhibition of angiogenesis by antibody blocking the action of proangiogenic high-molecular-weight kininogen. Journal of thrombosis and haemostasis : JTH 1(1):164-170.

**2. Supplementary figures**

**
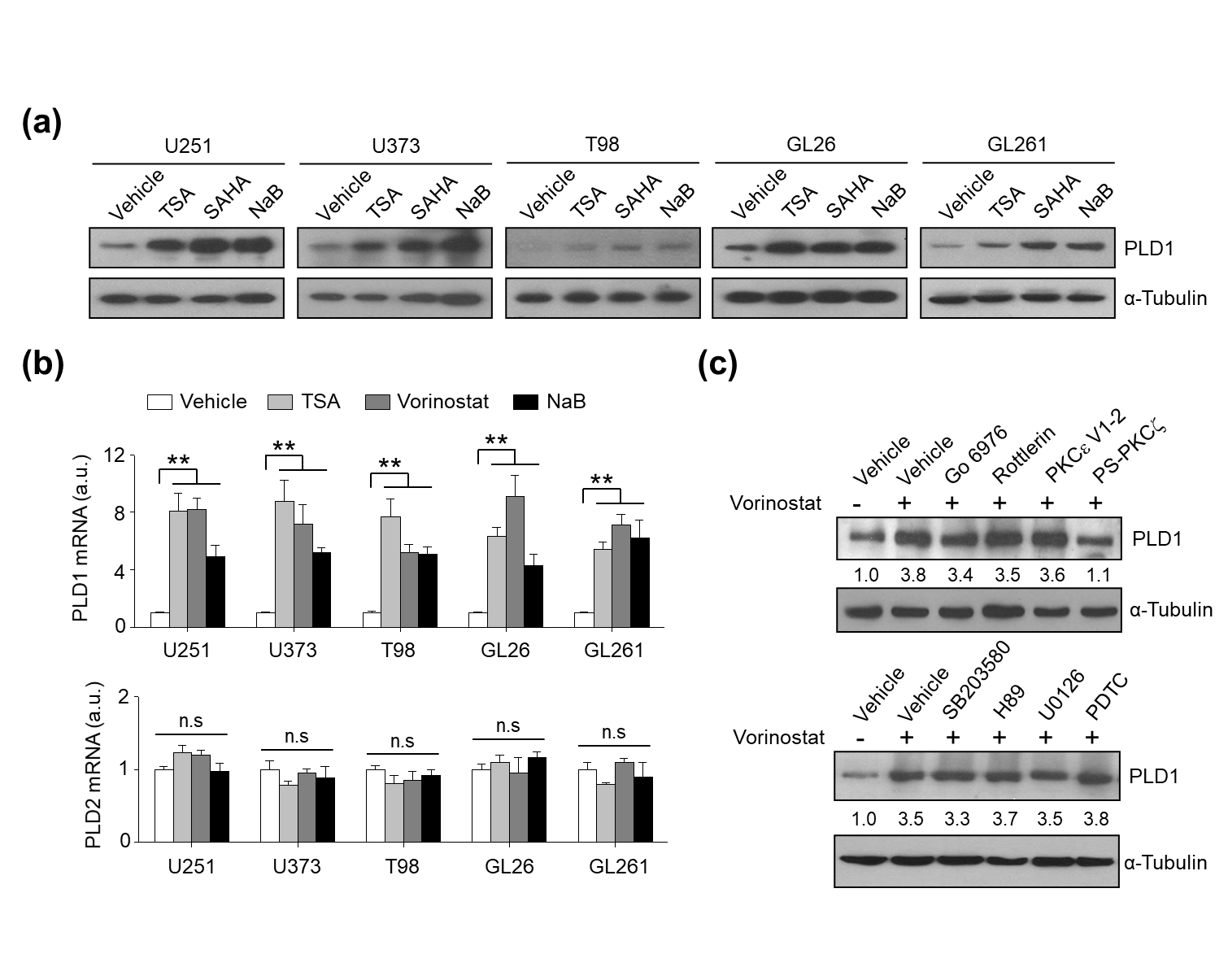
**

**Figure S1** Vorinostat increases expression of PLD1 via PKC** **The cells were treated with TSA (400 nM), SAHA (2 μM), and NaB (5 mM) for 24 hr, and expression of the indicated genes was analyzed by western blot (a) and q-PCR (b). (C) U87 cells were pretreated with the indicated inhibitors for 30 min and then treated with SAHA for 24 h. PLD1 expression was analyzed by western blot. Results are representative of at least three independent experiments and shown as the mean ± SEM. **p < 0.01; n.s, non-significant.

**
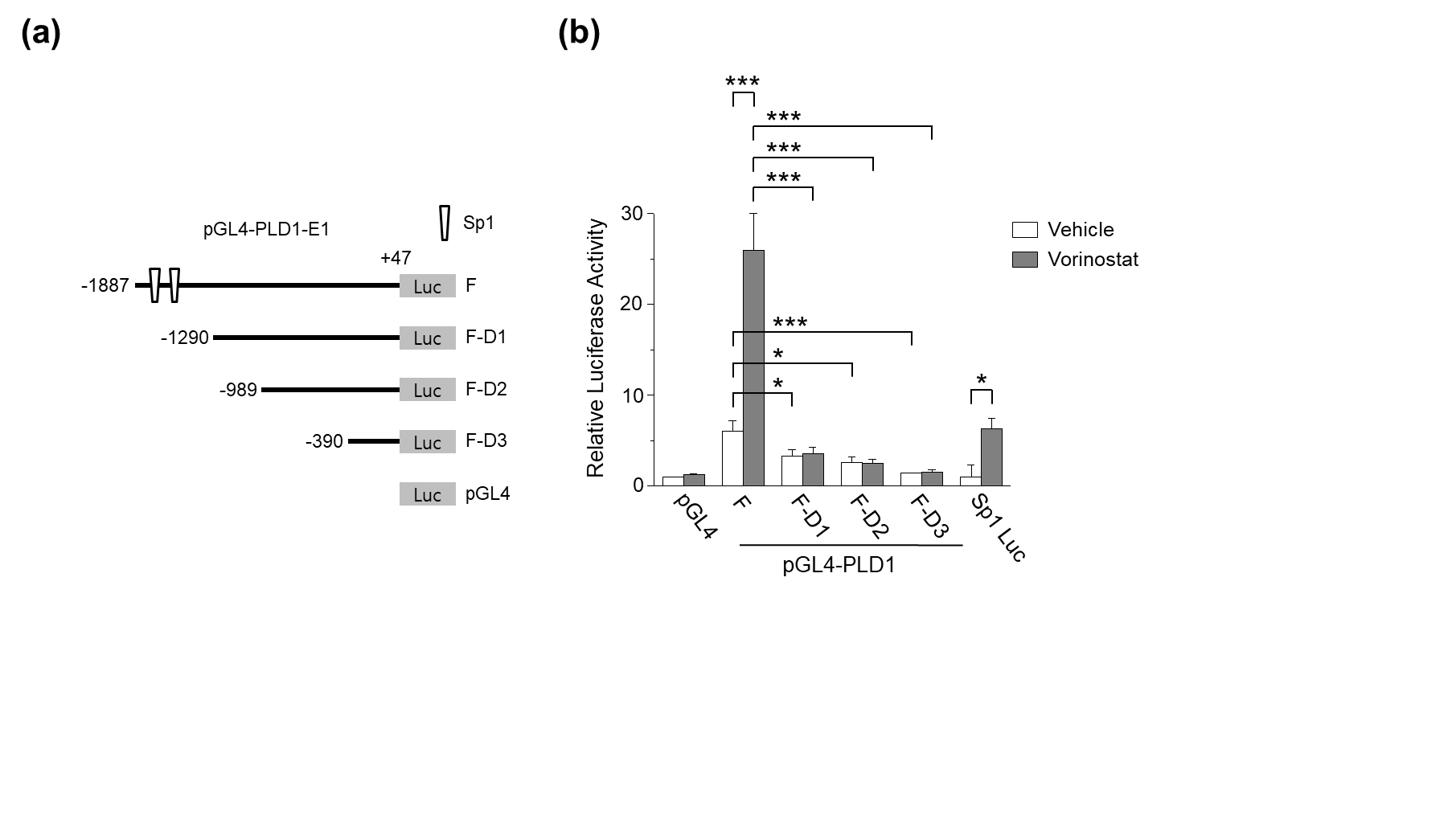
**

**Figure S2** The reporter gene assay using a series of 5΄-deletion constructs of the *PLD1* promoter.(a) Schematic representation of 5΄-deletion constructs of the *PLD1* promoter containing putative Sp1 binding sites. (b) Effect of vorinostat on the promoter activity of *PLD1* (right panel). Results are shown as the mean ± SEM. *p < 0.05; ***p < 0.001

**
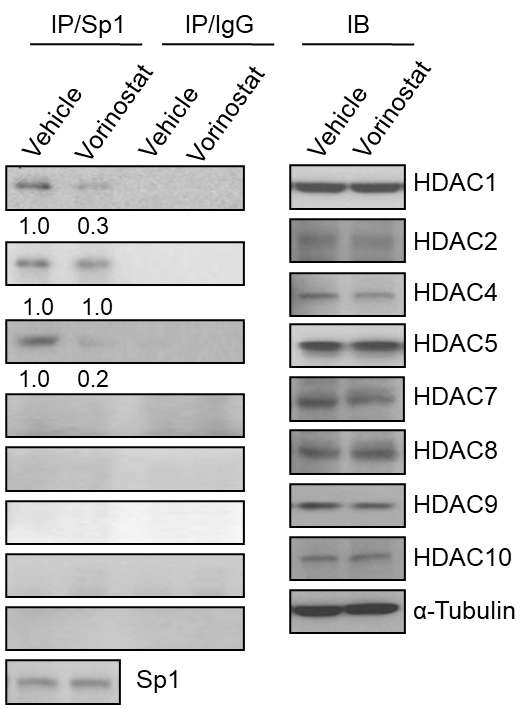
**

**Figure S3** Vorinostat decreases the interaction of Sp1 with HDAC1 and HDAC4.U87 cells were treated with vehicle and vorinostat, and the lysates were analyzed by immunoprecipptation and/or immunoblot using the indicated antibodies. Results are representative of at least three independent experiments.

**
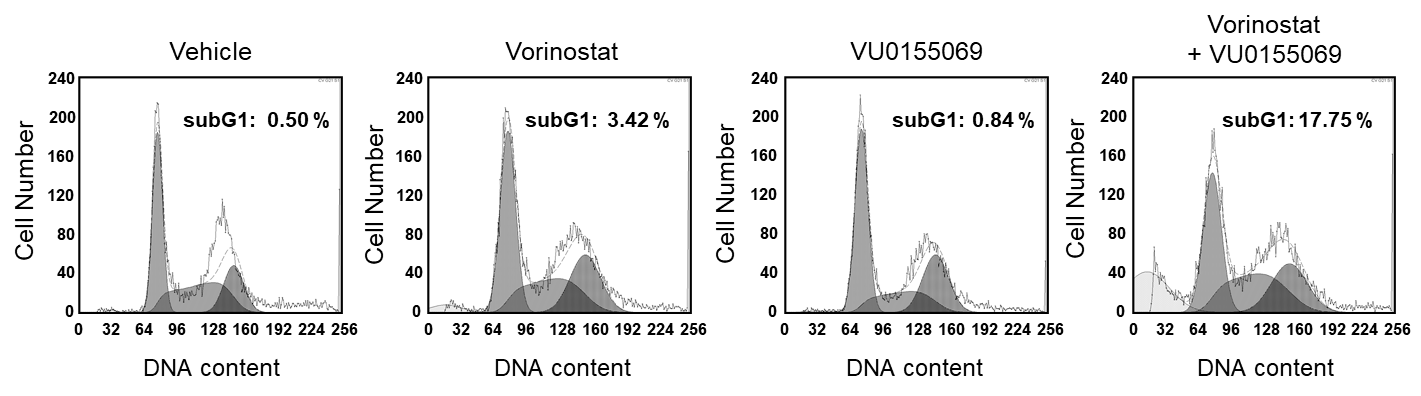
**

**Figure S4** Combination of vorinostat and PLD1 inhibitor increases the population of subG1 apoptotic cells.U87 cells were treated with vorinostat and/or PLD1 inhibitor, and analyzed by flow cytometry. Results are representative of at least three independent experiments.

**
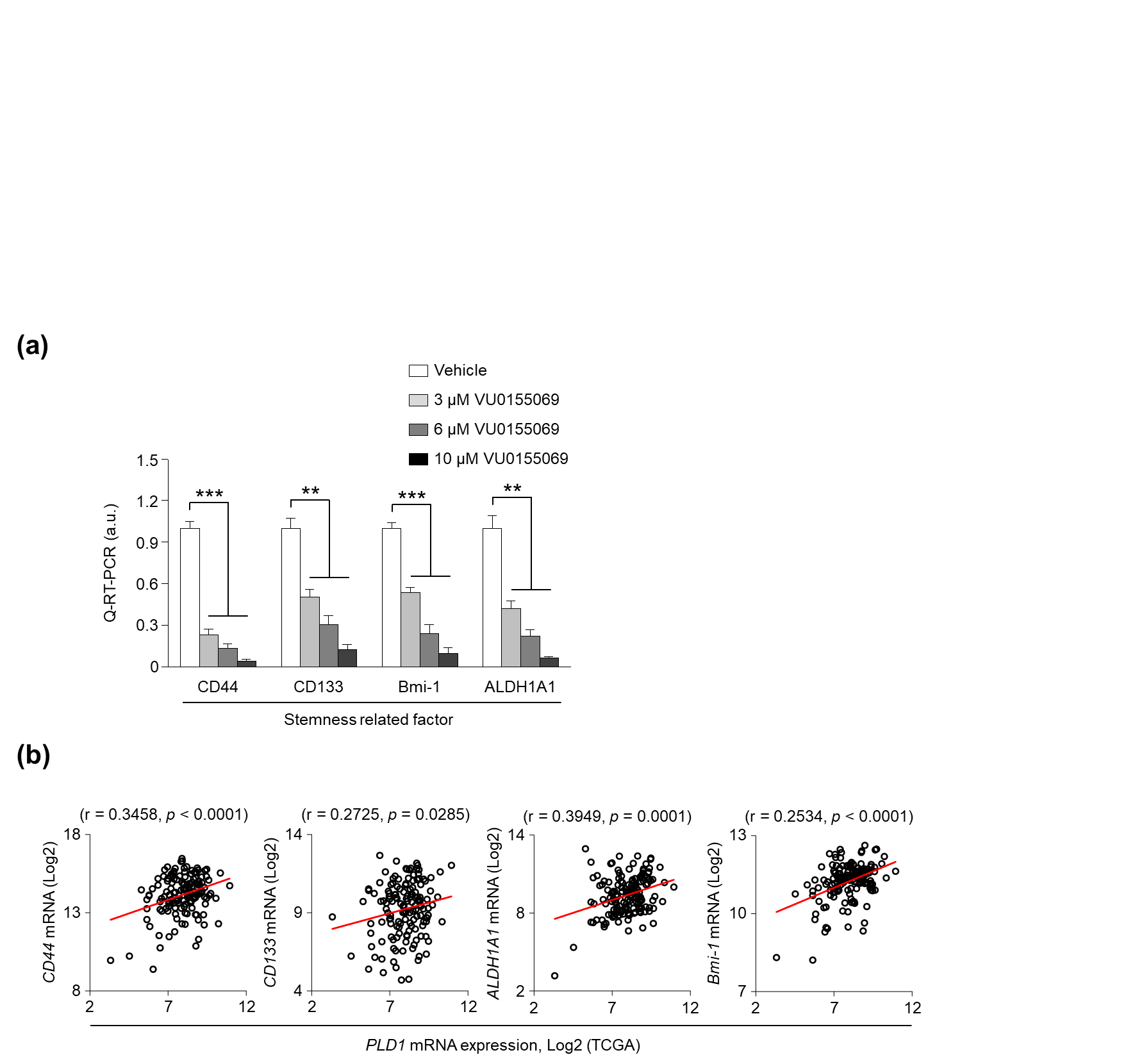
**

**Figure S5** PLD1 inhibition suppresses expression of stemness-related factors.(a)GBM-MES-83 cells under sphere culture condition were treated with the indicated concentration of PLD1 inhibitor. The expression of stemness related genes was analyzed by q-PCR. Results are shown as the mean ± SEM. **p < 0.01; ***p < 0.001.(b)The correlation of expression of the indicated genes with PLD1 mRNA levels was examined in TCGA GBM database. Spearman’s correlation coefficient (r) is provided along with its statistical significance. The red lines represent the best-fit curves
